# Inconclusive evidence for causal effects between rumination and sleep problems: a simulated reanalysis and comment on Yang and Lei (2025)

**DOI:** 10.3389/fpsyg.2025.1609290

**Published:** 2025-12-09

**Authors:** Kimmo Sorjonen, Bo Melin

**Affiliations:** Department of Clinical Neuroscience, Karolinska Institutet (KI), Stockholm, Sweden

**Keywords:** contradicting support, cross-lagged panel model, rumination, simulation, sleep problems, triangulation

## Abstract

**Background:**

Based on findings from analyses with cross-lagged panel models, Yang and Lei suggested reciprocal causal effects between rumination and sleep problems. However, it is well known that findings from cross-lagged panel models may be spurious.

**Method:**

We simulated data to resemble the data used by Yang and Lei. We used triangulation and fitted complementary models to the simulated data.

**Results:**

We found contradicting increasing and decreasing effects of initial rumination on subsequent change in sleep problems and vice versa.

**Conclusion:**

The divergent findings indicated that it is premature to assume causal effects between rumination and sleep problems and the suggestions by Yang and Lei in this regard can be challenged. It is important for researchers to be aware that correlations, including adjusted cross-lagged effects, do not prove causality in order not to overinterpret findings, something that appears to have happened to Yang and Lei. We recommend researchers to triangulate by fitting complementary models to their data in order to evaluate if analyzed data could be used to support contradicting conclusions, in which case the data should not be used to support any of those conclusions.

## 1 Introduction

Yang and Lei (2025) analyzed longitudinal data (two waves of measurement in 2019 and 2021, respectively) on rumination, i.e., repetitive thinking about causes and consequences of negative events, and sleep problems in a sample of university students in China (*N* = 373, mean age = 18.7 years (*SD* = 0.8 years), 77% females) with cross-lagged panel models. Yang and Lei (2025) reported statistically significant positive cross-lagged effects of initial rumination on subsequent sleep problems when adjusting for initial sleep problems and vice versa. Yang and Lei (2025) interpreted these findings in causal terms (e.g., in the title of their article).

However, it is well established that adjusted cross-lagged effects may be spurious due to correlations with residuals and regression to the mean (Campbell and Kenny, 1999; Castro-Schilo and Grimm, 2018; Sorjonen et al., 2019). For example, there appears to be a positive correlation between self-reported rumination and sleep problems (Yang and Lei, 2025), which could be due to a confounding impact by a third variable, e.g., general positivity. Therefore, we should expect that among individuals with the same initial score on sleep problems, those with a higher rumination score have received a lower score on sleep problems compared with their true sleep problems, i.e., a more negative residual, while those with a lower rumination score have received a higher sleep problems score compared with their true score, i.e., a more positive residual. However, residuals tend to regress towards a mean value of zero between measurements. Consequently, we should expect a more positive, but spurious, change in the sleep problems score to a subsequent measurement among those with a higher initial rumination score compared with those with the same initial sleep problems score but with a lower initial rumination score.

For the sake of argument and in agreement with Yang and Lei (2025), let us assume that a positive effect of initial rumination on subsequent sleep problems when adjusting for initial sleep problems can be interpreted as a causal increasing effect (not an interpretation we endorse). Then, for consistency, a positive effect of initial rumination on initial sleep problems when adjusting for subsequent sleep problems could be interpreted as a causal decreasing effect. This positive effect would suggest that high initial rumination had decreased high initial degree of sleep problems to the same subsequent level as among individuals with lower initial degree of sleep problems and lower initial degree of rumination. Moreover, a positive, a negative, and a null effect of initial rumination on the subsequent - initial sleep problems difference score could be seen to suggest an increasing, a decreasing, and no causal effect, respectively.

We estimated the effects outlined above, as well as the reversed effects of sleep problems on rumination, in data simulated to resemble the empirical data used by Yang and Lei (2025), with the same sample size and correlations between variables (these correlations are included in our analytic script available at the Open Science Framework, see link below). We used simulated data as the empirical data were not available to us. The corresponding author of the study by Yang and Lei (2025) did not respond to our request for the data. It should be noted that both the standardized effect of X on Y_2_ when adjusting for Y_1_ (Equation 1, Cohen et al., 2003) and on the Y_2_-Y_1_ difference (Equation 2, Guilford, 1965) are functions of correlations between the variables. Consequently, these effects will be the same in data, empirical or simulated, with the same correlations between variables and simulated data can be used to estimate what the effects would have been in corresponding empirical data.

Our simulated data did, by design, satisfy assumptions of regression analyses, e.g., normally distributed residuals and lack of influential outliers. We do not know if this was the case for the empirical data used by Yang and Lei (2025) as well. However, we do not believe that it would be tenable to argue that due to potential violation of regression assumptions in the data used by Yang and Lei (2025), we should trust their conclusion of causal increasing effects between rumination and sleep problems and ignore our warning (see below) against causal inference.

Analyses and the simulation for the present study were conducted with R 4.4.0 statistical software (R Core Team, 2025) using the MASS package (Venables and Ripley, 2002). The analytic script, which also generates the simulated data, is available at the Open Science Framework at https://osf.io/cwx7s/.


$$E(\beta_{X,Y2.Y1})=\frac{r_{X,Y2}-r_{X,Y1}r_{Y1,Y2}}{1-r_{X,Y1}^{2}}$$ (1)

$$E(\beta_{X,Y2-Y1})=\frac{r_{X,Y2}-r_{X,Y1}}{\sqrt{2(1-r_{Y1,Y2})}}$$ (2)

Initial rumination had a positive effect on subsequent sleep problems when adjusting for initial sleep problems (*β* = 0.198 [0.085; 0.312], *p* < 0.001) and initial sleep problems had a positive effect on subsequent rumination when adjusting for initial rumination (*β* = 0.163 [0.049; 0.277], *p* = 0.005). These effects could be seen to suggest that initial rumination had an increasing causal effect on subsequent change in sleep problems and vice versa. However, initial rumination also had a positive effect on initial sleep problems when adjusting for subsequent sleep problems (*β* = 0.496 [0.408; 0.584], *p* < 0.001) and initial sleep problems had a positive effect on initial rumination when adjusting for subsequent rumination (*β* = 0.494 [0.407; 0.581], *p* < 0.001). These effects could be seen to suggest, paradoxically, that initial rumination had a decreasing causal effect on subsequent change in sleep problems and vice versa. Moreover, initial rumination had a negative effect on the subsequent-initial difference score on sleep problems (*β* = −0.199 [−0.299; −0.099], *p* < 0.001) and initial sleep problems had a negative effect on the subsequent-initial difference score on rumination (β = −0.218 [−0.317; −0.118], *p* < 0.001). These effects could be seen to suggest that initial rumination had a decreasing causal effect on subsequent change in sleep problems and vice versa. According to proposed benchmarks (Orth et al., 2022), all effect sizes reported above were large (|β| ≥ 0.12).

In summary, the present findings showed that the data analyzed by Yang and Lei (2025) could be used to support contradicting conclusions of increasing and decreasing effects of initial rumination on subsequent change in sleep problems and vice versa. Hence, the data cannot support any of those conclusions and the conclusions of causal increasing effects by Yang and Lei (2025) appear premature and questionable. It is important for researchers to bear in mind that correlations, including adjusted cross-lagged effects, do not prove causality in order not to overinterpret findings, something that appears to have happened to Yang and Lei (2025). We recommend researchers to, as we did here, use triangulation by fitting complementary models to their data. If results from the models converge, conclusions of causality are corroborated (although never finally proven). If, on the other hand and as in the present case, findings diverge, conclusions of causality would appear premature. It should be noted that here, we do not use the term “triangulation” in a broad sense, involving analyses of multiple data sources, multiple theoretical perspectives, etc. Rather, in line with some recent studies (e.g., Sorjonen et al., 2025a; Sorjonen et al., 2025b; Sorjonen and Melin, 2024, 2025a, 2025b), we use the term to refer to fitting different statistical models to the same data.

## Data Availability

Publicly available datasets were analyzed in this study. This data can be found at: the analytic script, which also generates the simulated data, is available at the Open Science Framework at https://osf.io/cwx7s/.
